# Supplementary material for: Are carbon emissions trading and green financial instruments synergistic? -Comprehensive quantitative research based on content analysis
Source: PLoS One. 2024 Mar 7;19(3):e0298601. doi: 10.1371/journal.pone.0298601 (PMC10919601; doi:10.1371/journal.pone.0298601)
Supplement: S1 Data — (ZIP) [file pone.0298601.s001.zip › Data/地级市Z_F工作报告.zip!/─┌├╔╣┼/░═╤σ─╫╢√╩╨/└┤╫╘╣1⁄2╓┌║┼í╛┬φ┐╦╩2╛▌═°í┐_2018.docx]

各位代表：

　　现在，我代表市人民政府向大会报告工作，请予审议，并请市政协委员和列席会议的同志提出意见。

　　一、过去五年的工作

　　过去五年是我市发展进程中极不平凡的五年。面对经济发展进入新常态等一系列深刻变化，在市委的正确领导下，我们认真贯彻党中央国务院、自治区党委政府的各项决策部署，坚持稳中求进工作总基调，迎难而上、开拓进取，推动经济社会发展迈上了新台阶，为全面建成小康社会奠定了坚实基础。

　　——综合实力稳步提升。剔除虚增因素，预计地区生产总值年均增长7.2%，固定资产投资年均增长8.5%，社会消费品零售总额年均增长10.6%，一般公共预算收入年均增长2.9%，城乡居民人均可支配收入年均分别增长8.9%、9.8%。在经济稳步发展的基础上，我们着力补短板、惠民生，增强了各族群众的获得感、幸福感。

　　——产业结构优化升级。粮食生产能力稳定在60亿斤以上，肉羊饲养量和出栏量均居全区第一，有机原奶产量居全国首位。巴美肉羊新品种培育成果荣获国家科技进步二等奖。农畜产品加工转化率由69%提高到76%，新能源、新材料、大数据等新兴产业迅速发展，风光电装机占全市电力总装机的比重达64%，多晶硅产能占全区的40%。第三产业对生产总值增长的贡献率达到61%，提高了44个百分点。新增国家4A级旅游景区5个，黄河河套文化旅游区、纳林湖等景区知名度不断提升，富强村被评为“中国十大最美乡村”。电商、金融、物流等现代服务业蓬勃发展。

　　——城乡环境大为改观。城镇建成区面积达到128.6平方公里，常住人口城镇化率达到54.2%，城乡规划建设管理工作走在全区前列。新增公路里程2543公里，总里程和路网密度均居全区第三位，行政嘎查村全部通沥青水泥路。取消临陕公路收费站，促进了两地同城化发展。灌区续建配套与节水改造、排水设施改造、高标准农田建设成效显著，近70%的耕地生产条件得到改善。完成林业生态建设410.8万亩，套区森林覆盖率达到20%，新增湿地10.4万亩，成为国家生态文明建设先行示范区，荣获国土资源节约集约模范市。

　　——改革开放深入推进。在经济、生态和社会事业领域形成改革成果547个。习近平总书记嘱托的三项重点改革成效初显，供给侧结构性改革有效落实，“放管服”改革不断深化。农村牧区综合改革稳步推进，土地草牧场确权进展顺利。教育医疗、财税金融、生态文明等改革有序实施。全方位对外开放新格局初步形成，对蒙合作更加紧密。保税物流园区陆港通关运营，农畜产品出口额连续9年居全区首位，累计引进国内到位资金1473亿元。

　　——人民生活持续改善。各级财政累计投入民生资金816.1亿元，占一般公共预算支出的70%以上。9.92万名贫困人口稳定脱贫，贫困发生率下降到0.5%。就业形势保持稳定，覆盖城乡居民的社会保障体系基本建立，保障标准达到或超过全区平均水平，建成养老院、日间照料中心等养老服务设施48个，36万户城乡中低收入家庭居住条件得到改善。各级各类教育协调发展，十二年免费教育全面实施。市医院、市中医院晋升为国家三甲医院，市医院新址建成投用。公共文化服务体系日益完善，河套文化的影响力传播力显著增强。平安巴彦淖尔建设扎实推进，群众安全感明显提升。荣获全国双拥模范城、自治区文明城区。

　　——自身建设不断加强。认真履行全面从严治党责任，严格执行中央八项规定、国务院“约法三章”，深入开展党的群众路线教育实践活动、“三严三实”专题教育和“两学一做”学习教育，政府党组和政府系统党的建设进一步加强。主动接受人大依法监督和政协民主监督，建议和提案办复率均达到100%。积极配合人大做好立法工作，《河套灌区水利工程保护条例》《乌梁素海自治区级湿地水禽自然保护区条例》公布实施。“六五”普法圆满完成，“七五”普法有序开展，市县乡三级政府普遍建立了法律顾问制度。审计、监察工作力度加大，政府系统廉政建设深入开展。

　　各位代表，刚刚过去的2017年，是大事多、要事多、喜事多、难事也多的一年。我们成功组织了庆祝自治区成立70周年系列活动，举办了长三角、京津冀、珠三角大型招商引资推介会，承办了全国农村产业融合发展现场会、全国农商互联产销对接会、全区电子商务进农村牧区工作现场会、内蒙古绿色农畜产品国际博览会、全区综合行政执法改革现场会等重大活动。我们以高度的政治自觉，推进中央、自治区巡视反馈问题和环保督察反馈意见整改工作，积极化解政府债务和防范财政金融风险。

　　我们坚持长痛不如短痛，下决心挤掉多年积累的经济数据水分，并对政府投资类项目进行全面梳理，坚决砍掉过度超前、盲目举债投资的项目，坚决叫停不直接推动产业发展、不直接服务和改善民生的项目，已开工的合理“瘦身”。预计全年地区生产总值完成820亿元，剔除2016年虚增部分实际增长4.8%；一般公共预算收入完成57.2亿元，剔除2016年虚增空转因素，实际增长1.7%，非税收入比重由2016年30.6%下降至21.9%；社会消费品零售总额完成278亿元，增长7.9%；城乡居民人均可支配收入分别达到28255元、15634元，增长7.6%、8%，高于经济增速。综合分析各方面情况，我市经济运行的基本面是好的，预计全年工业用电量增长17.6%，铁路货运量增长15%，信贷投放量增长13.3%，规模以上工业企业盈亏相抵后利润增长51%，单位GDP能耗、主要污染物排放量持续下降。经济运行稳中有进、市场预期向好。更重要的是，我们放下了困扰多年的“泡沫”包袱，为轻装上阵、扎实前进奠定了基础。

　　一年来我们主要做了以下工作：

　　一是把项目建设作为突破口，不断增强经济发展后劲。深入开展“项目建设年”活动，开复工亿元以上重点项目121项。东立多晶硅一期、恒嘉蓝宝石一期、华拓矿业节能改造、金鼎钙白粉、美洋洋肉羊精深加工、仁创科技高密度循环水养殖、大漠风电5万千瓦供热、磴口500千伏变电站等项目建成投产。神华国能20万千瓦风电、龙腾10万千瓦光热、中粮集团30万吨优质面粉加工、康斯特4500吨果胶等项目加快推进。包银高铁完成线位选择和站场选址，京新高速巴彦淖尔段、省道311建丰农场至建设段、青山至乌根高勒一级公路、沿黄一级公路九庄段建成通车。制定出台招商引资优惠政策，与首农、西贝、中国金融租赁、中交疏浚、国电投等企业签订合作协议，力景华荣生物有机肥、天衡制药等项目落地建设，天津城投供排水一体化、五原县万亩现代农业示范园等PPP项目顺利实施。

　　二是把建设河套全域绿色有机高端农畜产品生产加工输出基地作为目标，全面深化农牧业供给侧结构性改革。大力发展绿色、生态、高效农牧业，新增设施农业2.3万亩，建成标准化规模养殖场170个，牧业年度牲畜饲养量达到2276万头（只）。新增“三品”产品81个，河套蜜瓜、河套西瓜、二狼山白绒山羊等6个产品获得农业部农产品地理标志登记保护，河套地域品牌美誉度不断提升。扎实推进农村牧区产业融合发展，农村电商、乡村旅游蓬勃兴起，农企利益联结比例达到75%，20万户农牧民进入产业化经营链条，产业融合发展工作走在全区前列。杭锦后旗入选国家农业可持续发展试验示范区和特色农产品优势区，磴口县获评国家有机产品认证示范创建区。

　　三是把稳定企业运行作为首要任务，促进工业经济提质增效。认真落实中央和自治区扶持企业发展的政策措施，加强工业经济运行调度，做好煤电油运等要素保障，促进企业稳定生产经营。全年减轻企业税费负担17.5亿元、用电成本5.4亿元，帮助中小微企业协调解决融资贷款263亿元。推动停限产企业恢复生产，停产和亏损企业分别减少6户、14户。投资5.7亿元，实施工业园区污水处理、管网配套、园林绿化等基础设施项目，综合承载能力进一步提升。

　　四是把发展现代服务业作为战略举措，加快培育新的经济增长点。实施黄河河套文化旅游区、五原抗战纪念园、乌拉特部落海洋馆等项目52个，黄河风情特色小镇、河套农耕文化博览苑入选全国优选旅游项目名录。成功举办国际马拉松赛、铁人三项赛、全国龙舟赛、沿黄河公路自行车赛、鸿雁文化艺术节、首届国际丝路骆驼文化旅游节、年猪文化节、冬捕节、花灯节、梨花节等活动，越来越多的中外游客慕名而来。全年接待游客552万人次，实现旅游收入56.2亿元，分别增长29.8%、30.1%。B型保税物流中心项目正式获批，陆港农副产品海铁班列开通。甘其毛都口岸过货量突破1800万吨，贸易额达到200亿元左右，成为全国过货量最大、贸易额最多的公路口岸。作为全国第九个网络市场监管与服务示范区，创建工作全面启动。电商企业达到1202家，增长11.1%；交易额实现41亿元，增长25.6%。金融环境进一步优化，金融机构存贷款余额分别达到967亿元和855亿元。

　　五是把改善人居环境作为工作重点，着力推进城乡建设。牢固树立民生城建理念，新改扩建城镇道路92公里、各类管网327公里，打通“断头路”19条，改造城镇平房区和老旧小区176万平方米、背街巷道60万平方米，新增集中供热面积480万平方米、天然气入户8621户、公厕131座。新增园林绿地3010亩，成功创建为国家园林城市，6个旗县区获评自治区生态宜居县城。双河区建设投入创历史新高，先锋桥、先锋南路、云中大街建成通车，市委党校新校区、内蒙古美术职业学院新校区、植物园等11个项目有序推进，“两桥两横两纵”路网主框架基本成型。县城和镇村管理水平不断提升，69%的村庄建立了长效管护机制，创建国家改善农村人居环境示范村和中国传统村落4个、自治区绿色村庄2566个。

　　六是把山水林田湖草沙综合治理作为总抓手，加大生态建设和环境保护力度。484万亩盐碱地改良工程得到国家和自治区高度重视，规划编制已完成，土壤普查工作稳步推进，开展试验示范2.1万亩。永久基本农田划定工作全面完成，划定面积807.9万亩。河长制得到落实，乌梁素海点源污水“零入海”和中水回用工程启动实施，36个水质监测点建成投用，生态补水2.5亿立方米。完成林业生态建设92万亩，发放草原生态补奖资金3.2亿元，乌兰布和沙区新增沙产业面积1.3万亩。巴彦淖尔生态治理与绿色发展院士专家工作站挂牌。持续开展生态环境保护大检查，一批群众反映强烈的环境问题得到整治。

　　七是把增进民生福祉作为工作出发点，努力让群众过上更加美好的生活。各级财政民生支出占一般公共预算支出的74.7%。扶贫支出5.6亿元，1.4万名贫困人口实现脱贫。新增城镇就业1.14万人，农牧民转移就业13万人次。社会保障实现人群全覆盖。开工建设棚户区改造5000套、农村牧区危房改造1287户。黄河水厂续建及配套工程竣工投运，中心城区居民喝上了优质放心的黄河水。政府还贷二级以下公路全部取消收费，投放新能源公交车53辆、共享单车2000辆。7个旗县区全部通过国家县域义务教育均衡发展验收，义务教育阶段学校“大通铺”问题得到全面解决。成立全区首家“医共体”。广电传媒中心建成入驻，电视播出迈入高清时代。乌拉特中旗乌兰牧骑荣获“全区十佳乌兰牧骑”称号，《鸿嘎鲁》获得全区首届蒙古语戏剧节入选剧目奖，足球主题公园建成开放。社会治安形势持续好转，刑事和“两抢”案件分别下降5.9%和53.8%，信访批次和人数分别下降9.1%和19.6%。安全生产事故起数和死亡人数实现“双下降”，食品药品安全形势总体平稳。

　　各位代表，五年来的奋斗充满艰辛，五年来的成绩有目共睹。这些成绩的取得，根本在于以习近平同志为核心的党中央坚强领导，在于习近平新时代中国特色社会主义思想的科学指引，得益于自治区党委、政府和市委的正确领导，得益于市人大、政协的有效监督与大力支持，得益于历任政府班子真抓实干、锐意进取，得益于全市各族干部群众攻坚克难、共同奋斗。在此，我代表市人民政府，向全市各族人民，向各位人大代表、政协委员和社会各界人士表示衷心的感谢！向所有为巴彦淖尔建设和发展作出贡献的同志们、朋友们，表示崇高的敬意！

　　各位代表，成绩属于过去，未来仍需努力。我们清醒地认识到，经济社会发展和政府工作中还存在一些突出问题、面临不少挑战。主要有：一是经济总量小，产业结构不合理。农牧业大而不强，很多产品好种不好卖、出产不出名、优质不优价；传统产业“原字头”企业多、产业链条短、产品附加值低，战略性新兴产业比重较小，非公有制经济发展不充分，全社会信用环境亟待改善；旅游业发展尚处于起步阶段，缺乏在全国全区有影响力的旅游景区；项目储备不足，大企业大项目偏少；创新能力不强，科技对经济增长的贡献率较低；口岸进口煤铜资源大多“穿肠而过”，就地加工转化率不高。二是生态建设和环境保护任重道远。破坏生态、污染环境的事件还屡有发生；矿山资源开发利用粗放，部分地区地下水超采严重；乌梁素海、乌拉特草原、乌兰布和沙漠、阴山山脉等重点生态功能区环境仍然脆弱，保护和治理任务艰巨。三是城乡基础设施比较薄弱。旧城区与新区基础设施还有不小差距，全市有1000多万平方米棚户区尚未改造，房地产开发还有很多遗留问题，有325万平方米“烂尾楼”“半拉子”工程；城市交通拥堵、停车困难、物业管理水平不高等问题逐步凸显；临河城区群众反映多年的北边渠改造问题还没有彻底解决；农村厕所、垃圾污水收集处理等设施不够完善，牧区通信、供电、供水保障能力不足。四是我市财力规模小，财政持续增收难度大，收支矛盾突出，政府债务已进入偿还高峰期，加之我们要在四年内将政府债务率降到合理水平，集中偿还压力较大。五是民生领域短板较多。城乡居民收入偏低，持续增收面临较大压力，乌梁素海周边、山旱区、边境牧区等地区发展滞后，部分群众生活还比较困难；养老、文化、体育等公共服务相对滞后，优质的教育、医疗资源不足，“择校热”“看病贵”等问题依然存在，农牧民工工资保障机制还不够完善；新老社会矛盾交织，一些重点领域、重点群体上访问题依然突出。六是政府自身建设还有很多不足。“四风”问题新表现在部分单位和干部身上不同程度存在，一些单位“门好进、脸好看、事难办”，服务意识不强、行政效能不高，少数领导干部为官不为、不敢担当，个别国家工作人员懒政怠政、不思进取；一些重点领域违规违纪现象仍有发生，党风廉政建设还需常抓不懈，等等。为此，我们要坚持问题导向，强化责任担当，以务实的态度直面问题，以创新的思路解决问题，努力把政府工作做得更好。

　　二、今后五年的工作

　　今后五年，是“两个一百年”奋斗目标的历史交汇期，也是我们实现塞上江南、绿色崛起的重要战略机遇期。党的十九大确立了习近平新时代中国特色社会主义思想的历史地位，提出了新时代坚持和发展中国特色社会主义的基本方略，描绘了决胜全面建成小康社会、夺取新时代中国特色社会主义伟大胜利的宏伟蓝图，为我们推动各项事业发展指明了前进方向。国家深入推进“一带一路”“中蒙俄经济走廊”建设，实施西部大开发、京津冀协同发展战略，支持民族地区、边疆地区和贫困地区改善生产生活条件，为我们提供了难得的发展机遇。新时代新征程要有新作为。我们要进一步增强机遇意识、责任意识、使命意识，努力用创新思维和方法开展工作，以更大的勇气和智慧开拓前行，在新的起点上谋划新发展、实现新突破。

　　今后五年政府工作的总体思路是：全面贯彻党的十九大精神，以习近平新时代中国特色社会主义思想为指导，统筹推进“五位一体”总体布局，协调推进“四个全面”战略布局，坚持稳中求进工作总基调，落实新发展理念，坚决打赢防范化解重大风险、精准脱贫、污染防治三大攻坚战，坚决守好发展、民生、生态三条底线，解放思想、开拓创新、真抓实干，推进高质量的发展，把巴彦淖尔建设成为绿色产业集聚区、生态文明示范区、乡村振兴样板区、对蒙开放先行区、美好生活共享区，努力建成现代化生态田园城市，实现塞上江南、绿色崛起。

　　经济社会发展的主要目标是：全市经济实力显著增强，生态环境更加优美，深化改革取得决定性成果，基础设施支撑保障能力进一步提升，群众生活更为宽裕，与全国全区同步全面建成小康社会，开启现代化巴彦淖尔建设新征程。

　　实现这一目标，必须付出更为艰巨的努力。从巴彦淖尔实际出发，我们要坚定不移走绿色发展之路，把绿色发展理念贯穿经济社会发展全过程、各领域，大力提升发展质量和效益，着力解决发展不平衡不充分的问题，更好满足人民群众日益增长的美好生活需要。

　　（一）坚持兴产业、促转型，构建绿色现代产业体系。发展是解决我市一切问题的基础和关键。我们要把推进绿色发展、高质量发展作为主攻方向，加快建设实体经济、科技创新、现代金融、人力资源协同发展的产业体系。大力实施乡村振兴战略。按照产业兴旺、生态宜居、乡风文明、治理有效、生活富裕的总要求，健全城乡融合发展体制机制，加快推进农牧业和农村牧区现代化。深化农牧业供给侧结构性改革，优化产业体系、生产体系和经营体系，推动“三区三园一体”建设，促进农村牧区产业融合发展。保障粮食安全，调优种养结构，发展精深加工，推行绿色生产，实施河套地域品牌战略，培育产业化联合体、新型经营主体、新型职业农牧民，建设河套全域绿色有机高端农畜产品生产加工输出基地，打造“塞外粮仓、天下厨房”“草原上的菜篮子、黄河边上的农艺园”。统筹抓好农村牧区基础设施、公共服务、精神文明、生态建设，让农牧业强起来、农牧区美起来、农牧民富起来。加快新型工业化进程。坚持提升传统产业与培育新兴产业并重，壮大骨干企业与发展中小微企业并举，加大项目建设力度，推动工业经济扩量提质增效。积极发展新材料、新能源、大数据运用、生物科技等产业，多晶硅产能达到5万吨，风光电装机达到1000万千瓦，建成国家清洁能源基地。认真落实主体功能区规划和产业指导目录，优化产业布局，实施园区振兴计划，套区工业园区重点发展农畜产品精深加工、高新技术等产业，沿山工业园区集中发展现代冶金、新型化工、新型建材等产业。发展壮大现代服务业。以建设河套文化特色旅游度假基地为目标，大力发展全域旅游、四季旅游、全要素旅游，形成黄河风情游、田园休闲游、草原观光游、沙漠探险游、中蒙跨境游等多种业态，新增国家4A级以上景区6个，把旅游业打造成为战略性支柱产业。做大做强现代物流、电子商务、健康养老等产业，力争服务业占生产总值的比重达到40%以上。深入推进创新驱动发展。加大科技创新投入力度，加强创新平台载体建设，发挥各类创新主体作用，推进产学研用深度融合。实施人才强市战略和“河套英才”工程，加快建设新型智库，用活用好人才，强化创新发展的人才保障。增强金融服务实体经济能力。大力发展普惠金融，提高直接融资比重，促进多层次资本市场健康发展。强化财税收支管理，提升财政保障能力。

　　（二）坚持修生态、筑屏障，加快建设美丽巴彦淖尔。幸福生活不只在于丰衣足食，也在于碧水蓝天。我们要统筹抓好山水林田湖草沙综合治理，推进河套平原绿化、乌梁素海综合治理、乌兰布和沙区治理、乌拉特草原保护、阴山山脉生态保护、黄河湿地保护等重大工程，积极参与沿黄生态走廊建设，基本完成484万亩盐碱地改良，全面提升生态系统质量和稳定性，套区森林覆盖率提高到21.5%，草原植被盖度达到25%。严格实行能源和水资源消耗、建设用地等总量和强度双控行动，认真落实节能降耗目标责任制，促进各类资源节约高效利用。倡导简约适度、绿色低碳的生活方式，开展创建节约型机关、绿色家庭、绿色学校、绿色社区和绿色出行等行动。

　　（三）坚持抓改革、扩开放，积极培育发展新动能。改革开放，是过去40年我们发展的最强音，也是今后实现绿色崛起的动力源。我们要以更加坚定的决心和勇气把改革进行到底，以更加宽广的视野和姿态全面扩大开放。以经济体制改革为重点，不断深化重要领域和关键环节改革，推出一批叫得响、立得住、群众认可的硬招实招，坚决破除一切不合时宜的体制机制弊端，打好全面深化改革攻坚战。主动融入和服务“一带一路”“中蒙俄经济走廊”建设，加强与京津冀、长三角、珠三角等地区合作，深度融入呼包银榆经济区。大力发展口岸经济，推动口岸功能区、口岸加工园区、B型保税物流中心联动发展，积极争取建设国家级重点开发开放试验区和中蒙跨境矿产能源合作试验区。理顺口岸管理体制，加强口岸规划建设管理，全力打造边境口岸特色城镇。培育壮大口岸进出口贸易、仓储物流、跨境旅游、跨境电商等产业，争取未来3年口岸年过货量突破5000万吨，资源就地精深加工能力得到大幅提升，人文交流更加密切，把甘其毛都口岸建设成为自治区新的经济增长极、对蒙开放桥头堡。

　　（四）坚持强基础、提品质，增强支撑保障能力。基础设施滞后是我市绿色发展的突出短板。我们要按照适度超前、合理布局、配套完善、适应发展的原则，完善交通、水利、电力、信息、市政、物流等重大基础设施，为推进绿色发展夯基垒台。推动建成包银高铁和乌拉特前旗、乌拉特后旗、磴口县通用机场，争取巴彦淖尔机场升级为4D机场，实现旗县区所在地、工业园区、口岸高等级公路全覆盖，完善现代综合交通运输体系。加强节水灌溉、饮水安全、防汛抗旱等水利工程建设，实现水资源合理高效配置。抓好城乡电网升级改造，推动电力外送通道建设，破解风光电外送瓶颈。加快推进智慧巴彦淖尔建设，建成覆盖城乡、高速畅通、技术先进的信息网络。稳步推进以人为核心的新型城镇化，坚持中心城区优先发展，带动县城和集镇建设，完善城镇道路、管网、供排水等基础设施，扩大基本公共服务供给，增强产业支撑和就业吸纳能力，加快农牧业转移人口市民化，提升城镇宜居宜业水平。

　　（五）坚持富百姓、增福祉，提高保障和改善民生水平。让巴彦淖尔老百姓的日子越过越好，是政府义不容辞的责任，也是发展的根本目的。我们要牢固树立以人民为中心的发展思想，既尽力而为，又量力而行，一件事情接着一件事情办，一年接着一年干，让人民群众生活幸福、社会和谐安宁。实施城乡居民增收计划，实现居民收入增长快于经济增长。坚持就业优先战略和积极就业政策，加强社会保障体系建设，织密织牢民生保障网。优先发展教育事业，办好各级各类教育，努力让每个孩子都能享有公平而有质量的教育。切实保障妇女儿童合法权益。加快建设健康巴彦淖尔，健全医疗卫生服务体系，为群众提供全方位全周期健康服务。完善公共文化服务体系，大力发展文化事业和文化产业，推动文化体育旅游融合发展，加强乌兰牧骑队伍建设，创作一批接地气、传得开、留得下的精品力作。充分发挥群团组织作用，开展群众性精神文明创建活动。深入推进依法治市，深化平安巴彦淖尔建设，落实党的民族和宗教政策，支持国防和军队建设，筑牢祖国北疆安全稳定屏障。

　　各位代表，从现在到2020年，是全面建成小康社会决胜期。行百里者半九十，离目标越近越要奋力前行。我们要突出抓重点、补短板、强弱项，特别是要坚决打好防范化解重大风险、精准脱贫、污染防治三大攻坚战。有效防范化解金融风险，坚决守住不发生系统性金融风险的底线；规范政府债务管理，积极化解存量债务，确保到2021年底将政府债务率降到合理水平。把提高脱贫质量放在首位，加大精准扶贫、精准脱贫力度，确保农村牧区贫困人口稳定脱贫。下大力气解决大气、水、土壤污染等环境突出问题，实现生态环境质量明显改善。我们要制定专项方案，拿出过硬举措，确保三大攻坚战取得突破性进展，使全面建成小康社会得到人民认可、经得起历史检验。

　　各位代表，人民对美好生活的向往就是我们的奋斗目标。我们坚信，只要全市上下勠力同心、脚踏实地、接续奋斗，就一定能够铸就巴彦淖尔新的辉煌，一定能够全面建成小康社会，一定能够实现塞上江南、绿色崛起！

　　三、2018年重点工作

　　2018年是贯彻党的十九大精神的开局之年，是改革开放40周年，是决胜全面建成小康社会、实施“十三五”规划承上启下的关键一年。我们要认真贯彻落实自治区党委十届五次全会暨全区经济工作会议、市委四届四次全会暨全市经济工作会议精神，努力做好今年的工作。主要预期目标是：地区生产总值增长6%，城乡居民人均可支配收入分别增长7.5%和8%；城镇新增就业1.1万人，城镇登记失业率控制在4%以内；单位地区生产总值能耗完成自治区下达的目标；政府债务化解完成总任务的23.6%，财政金融风险得到有效防控；供给侧结构性改革取得实质性进展。

　　以上主要预期目标，体现了稳中求进的要求，考虑了国家、自治区将继续调整经济指标和核算方法，也符合我市主动转换发展动能、优化产业结构的需要。地区生产总值按6%安排，略高于2017年实际增速，体现了更加注重提高发展质量和效益的导向；城乡居民人均可支配收入继续高于经济增长，体现了以人民为中心的发展思想；严格落实化解政府债务的刚性要求，增加了具体指标。需要强调的是，挤掉经济数据水分，淡化经济指标考核，不是“随大流”无所作为，而是为了放下包袱，放开手脚，靠实干兴业。我们一定要保持奋发有为的精神状态，力争把各项任务完成得更好。

　　实现上述目标，我们要重点抓好以下工作。

　　（一）坚决打好三大攻坚战。这是破解制约全面建成小康社会重点难点问题的现实需要。我们要切实提高政治站位，迎难而上、精准发力，确保三大攻坚战取得实效。

　　打好防范化解重大风险攻坚战。严格规范投资融资行为，持续开展打击非法集资行动和互联网金融风险专项整治，严厉打击违法违规金融活动。完善金融监测预警和应急处置机制，支持金融机构依法处置不良贷款。加强政府债务管理，通过预算安排、债权置换、资产重组、资源盘活等措施，多用市场化、金融等手段稳妥有序化解存量债务，不断降低政府债务率。强化财政约束，严格规范地方政府投资项目、举债融资、基金运作和购买公共服务行为，从严控制政府债务增量。完善政府举债终身问责制和债务问题倒查机制，加强审计监督和督查问责。从严控制政府债务、清理政府投资项目，不是不能上项目，而是杜绝过度举债、上不切实际的项目，那些发展所必须的、也有能力建设的项目，不仅要上，而且要加大力度建设。

　　打好精准脱贫攻坚战。坚持“三位一体”大扶贫格局，加大脱贫攻坚投入力度，瞄准特定贫困群众精准帮扶，对有劳动能力的贫困人口，采取产业扶贫、转移就业等办法实现脱贫；对丧失劳动能力的，落实救助供养、大病救治、生活兜底等保障措施，确保新识别和返贫的贫困人口全部脱贫，既不降低标准，也不吊高胃口，切实提高脱贫质量。在区域上，重点向乌梁素海周边、山旱区、边境牧区等贫困程度较深地区聚焦发力，在项目资金等安排上给予倾斜，着力改善贫困地区的发展条件。实施最严格的评估考核监督制度，开展扶贫领域作风问题专项整治，做到脱真贫、真脱贫。

　　打好污染防治攻坚战。严格落实国家《生态环境损害赔偿制度改革方案》，强化企业环境损害赔偿责任，破解“企业破坏、群众受害、政府买单”的难题。抓好中央和自治区环保督察反馈意见整改工作，按时限完成整改销号。深入实施蓝天行动计划，加快重点行业脱硫、脱硝及除尘升级改造工程建设，全面淘汰10蒸吨及以下燃煤小锅炉。加强与内蒙古环投、天津城投等企业合作，实施城镇和工业园区污水处理、中水回用等项目。推行垃圾分类制度，建设生活垃圾焚烧发电厂、餐厨垃圾处理厂。加大环保执法力度，坚决查处偷排偷放、超标排放等行为。

　　（二）全力建设河套全域绿色有机高端农畜产品生产加工输出基地。这是实施乡村振兴战略、促进城乡融合发展的核心和关键，也是深化农牧业供给侧结构性改革、提高农牧业质量效益和竞争力、促进农牧民持续增收的重要举措。我们要以强有力的工作措施，举全市之力推进。

　　抓好河套地域品牌建设。编制河套农畜产品品牌发展计划，制定完善扶持政策，完成“河套”地理标志商标注册，让“河套”成为巴彦淖尔绿色农畜产品的代名词。建立河套产品全程质量标准体系，鼓励引导企业、合作组织按照标准组织生产、加工和销售，对符合标准的产品授权使用河套地域品牌，让“河套牌”产品进入高端市场，卖上好价钱。强化农畜产品质量安全追溯管理，争取将肉类、果蔬、粮食等主要农产品全部纳入追溯体系。加大品牌宣传营销推介力度，提高河套地域品牌的知名度和影响力。积极培育特色农畜产品品牌，新增“三品一标”产品60个，做好市长质量奖评选工作，创建国家级出口食品农产品质量安全示范市。

　　调整优化农牧业结构。稳定粮食生产能力，调减籽粒玉米20万亩，优质小麦面积达到150万亩，葵花种植面积保持稳定，扩大优质牧草和小杂粮种植面积，鼓励有条件的旗县区因地制宜发展集中连片设施农业。稳羊增牛扩猪禽，适度发展肉驴、骆驼、水产等特色养殖，牲畜饲养量达到2400万头（只）。有序扩大经济林种植面积，积极发展林果业。

　　推进农村牧区产业融合发展。设立河套全域绿色有机高端农畜产品生产加工输出基地产业引导基金。培育和引进在全国全区有影响力的龙头企业10家以上，推进乳肉、粮油、绒纺等农畜产品精深加工，提升产业集中度和产业化水平。完善仓储、保鲜、烘干、清选分级等设施装备条件，促进农畜产品减损增效。壮大农村电商、订单直销等新业态，鼓励企业在大中城市设立农畜产品直销店，打造线上线下相融合的营销平台。培训农牧民30万人次，培育新型职业农牧民3000人。办好内蒙古绿色农畜产品国际博览会、首届内蒙古西部国际商贸展洽会。推进农牧业与旅游、文化等产业融合发展，每个旗县区建设一个田园综合体，力争临河区全域创建为国家农村产业融合发展示范园。

　　推广绿色集约安全生产模式。深入开展化肥农药零增长行动，推广控肥增效技术260万亩，增施有机肥370万亩。推广机械施肥、种肥同播、水肥一体、病虫害统防统治等技术，严禁使用高毒、高残留农药。在做好传统地膜回收基础上，引导农牧民使用高标准厚地膜，引进试验可降解地膜。规范限量使用饲料添加剂，减量使用兽用抗菌药。做好畜禽养殖废弃物资源化利用，抓好杭锦后旗、五原县种养业循环一体化整县推进试点工作。

　　（三）加快推进山水林田湖草沙综合治理。这是我们推动高质量发展、建设现代化生态田园城市的重要基础和保障。我们要把山水林田湖草沙作为生命共同体，用系统思维谋划和推动生态修复、综合治理、保护开发等工作，努力建设生态文明示范区。

　　加强市级层面统筹协调，制定山水林田湖草沙生态系统保护、修复和治理规划，设立综合治理产业基金，统筹推进重点工程建设。一是保护好山。抓好矿山地质环境恢复和综合治理，推动国家级绿色矿山建设。加大国家和自治区级自然保护区生态修复力度，依法严厉打击私挖乱采等违法行为。二是治理好水。大力发展节水灌溉，控制发展高耗水工业。建立地下水取用水总量和水位控制制度，严禁超采区新增取用地下水。三是营造好林。实施天然林保护、退耕还林、京津风沙源治理等重点生态工程，完成林业生态建设90万亩，优化造林结构，加大经济林比重。四是管护好田。严格落实耕地保护制度，禁止占用基本农田。以五原县5万亩示范园建设为重点，加快实施盐碱地改良工程，全市实施改盐增草兴牧重点区域30万亩，力争达到50万亩。开展粮食生产功能区划定工作，推进千亿斤粮食增产项目建设，完成高标准基本农田建设126万亩、耕地深松旋耕60万亩、秸秆还田180万亩。五是改善湖泊生态功能。推进乌梁素海水环境综合治理与可持续发展试点工作，抓好点源污水“零入海”工程，所有旗县区和巴彦淖尔经济技术开发区都要建成再生水厂，推进工业园区和企业废水循环利用，中水回用率达到60%以上。完成生态补水2.5亿立方米。六是守护好草原。开展“大美草原守护行动”，落实草原生态补奖政策，推行草原禁牧休牧轮牧和草畜平衡制度，促进草原生态环境持续好转。七是治理好沙。推进乌兰布和沙区治理，启动建设国内一流、功能多元、优势明显的沙产业园、沙生植物园，新增沙产业面积1.5万亩。

　　（四）扩大精准有效投资。今天的有效投资就是明天的生产力，抓项目、扩投资对促进我市经济社会发展具有决定性意义。我们要坚持多元投资、精准投资，激发民间投资活力，用好多层次资本市场，充分调动市场主体投资积极性。

　　抓好重点项目建设。全年实施亿元以上重点项目139项。完成交通投资30亿元，开工建设包银高铁，建成国道242甘其毛都至临河一级公路，推进甘其毛都经海流图至乌拉山一级公路、110国道临河至磴口段一级公路改造、国道242临河过境一级公路、国道335乌不浪口至乌根高勒二级公路、国道331乌珠尔至北银根三级公路等项目建设。完成水利投资20亿元，抓好灌区续建配套与节水改造、小型农田水利、高效节水灌溉等项目建设。完成电力投资17.9亿元，实施新安220千伏、永乐110千伏等48项输变电工程。统筹推进产业发展、生态环保、城乡建设和民生改善等领域项目。

　　加大招商引资力度。深入开展“招商引资年”活动，围绕山水林田湖草沙综合治理、农畜产品精深加工、战略性新兴产业、口岸经济等重点领域，开展一对一、点对点精准招商，引进一批实力强、技术新、信誉好的龙头企业。完善重大项目库建设，做好项目前期工作。持续跟踪对接招商项目，提高项目履约率和资金到位率，力争年内引进国内到位资金275亿元。

　　优化营商环境。营商环境就是竞争力、就是生产力。要深入开展营商环境专项整治行动，下大力解决“管卡压”“推绕拖”等突出问题，解决“门好进、脸好看、事难办”问题，解决重承诺、轻落实问题。加强信用信息平台建设，加大失信惩戒力度。实施市场准入负面清单制度，破除歧视性限制和各种隐性障碍，支持非公有制经济发展，激发和保护企业家精神，让各类市场主体在巴彦淖尔受到尊重、感到公平、成就事业。

　　（五）推动工业经济转型升级。突出工业主导地位，以重点项目建设为抓手，以工业园区为载体，推动工业经济高质量发展。

　　改造提升传统产业。运用新技术、新工艺和新装备，推动农畜产品加工、矿山冶金、能源化工等产业延链补链，提高附加值。加快实施黑猫煤化工、神华煤化工一期二线、海明110万吨铁精粉、金峰化工5亿立方米煤制气、旭一牧业3万头肉牛加工、润海源肉鸡产业化开发等项目，开工蒙驼骆驼产业、花麒驴产业、鸿景思德甜菜制糖、三聚绿能农作物秸秆综合利用等项目，积极推进白银集团铜冶炼项目前期工作。瞄准市场需求，发展壮大食品加工业，开发更多成品、半成品方便休闲食品。大力振兴羊绒产业，协调金融机构，纾解羊绒企业困境，促进行业内优化重组。

　　培育壮大新兴产业。加快杭龙生物质热电联产、四通风机制造、天衡制药等项目建设，推动中兴能源燃料乙醇、盾安多晶硅三期、东立电池组件二期、恒嘉蓝宝石二期、红田光电蓝宝石、广东富源蓝宝石等项目建成投产。启动丰域烯碳、金彩矿业石墨新材料等项目建设，筹建新材料产业联盟。积极争取国家光伏发电领跑者基地项目，推进百万千瓦风电基地建设，实施四华20万千瓦风电、蒙能10万千瓦风电、龙腾10万千瓦光热、北方联合电力5万千瓦光热等项目，新增风电装机90万千瓦、光伏50万千瓦。抓好乌拉特前旗启翔、乌拉特后旗算力、五原县创客等13个云计算项目，新增服务器20万台。积极推进中国电科智慧巴彦淖尔项目，推动大数据云计算广泛应用。

　　加强工业经济运行调度。抓好企业用电量、货运量、产品价格、利润税收等指标监测预测，强化精准调度。开展“走企业、解难题”活动，帮助企业协调解决信贷、土地、用工等问题，促进企业满负荷生产。认真落实国家和自治区税费减免、电价优惠等政策，下大力气减轻企业负担。探索建设微电网、清洁能源供暖等模式，提高本地电力消纳能力。进一步调整优化园区空间布局和产业规划，完善园区道路、供排水、电力等基础设施，大力推进专业化招商，探索“园中园”“飞地经济”模式，提高园区集约化发展水平。力争五原工业园区晋升为自治区级工业园区。

　　（六）提升服务业发展水平。适应消费升级新趋势，积极发展旅游、金融、电商物流等现代服务业，推动服务业发展提速、比重提高。

　　大力发展全域旅游、四季旅游。推动黄河河套文化旅游区、黄河风情特色小镇、五原抗战纪念园、纳林湖等景区提档升级，力争乌兰布和沙漠旅游区创建成为国家4A级旅游景区。完善旅游公路、旅游标识、自驾游营地、景区厕所等服务设施。深入开展旅游区域合作，开通哈尔滨——巴彦淖尔——博鳌季节性航线，争取开通中蒙自驾游线路。办好龙舟赛、沿黄河公路自行车赛、乌梁素海冬季冰雪旅游节、沙漠国际游牧音乐节、花灯节、梨花节等活动，力争接待游客660万人次以上，实现旅游总收入67亿元以上。

　　推动金融业健康发展。鼓励金融机构增加信贷投放，加大对小微企业、“三农三牧”、贫困地区的信贷支持力度，力争信贷总量达到1200亿元以上，社会融资规模增加220亿元以上。大力发展多层次资本市场，新增10家企业进入股权债权交易市场，累计直接融资突破百亿元。积极引进银行、保险、担保等金融机构，推动磴口县、乌拉特中旗、乌拉特后旗农村信用社改制为农商行。

　　加快发展电商物流业。推进全国网络市场监管与服务示范区创建工作，营造开放规范、安全放心、公平有序的网络环境。筹建全市统一的农畜产品销售电商平台，启动运营河套电商谷，实现旗县区电商产业园全覆盖，电商交易额突破50亿元，创建国家电子商务示范城市。推进快递下乡和电子商务进农村牧区，完善物流配送体系，实现规模50户以上的村组电商服务网点全覆盖。开通巴彦淖尔中欧农产品班列并逐步常态化，B型保税物流中心功能设施基本建成。

　　（七）深化改革扩大开放。改革开放只有进行时，没有完成时。我们要以深化改革破解发展瓶颈，以扩大开放拓展发展空间，激发经济发展的动力和活力。

　　推进习近平总书记嘱托的三项重点改革。全面推广订单合同型、股份合作型、服务协作型等紧密型农企利益联结模式，保障农牧民分享更多增值收益。做好生态保护红线划定工作。出台河套灌区管理体制改革意见，理顺国管群管水利体制，推进农业水价综合改革。坚持和深化河长制，实施湖长制。创新对蒙合作机制，协调蒙方在我市设立领事分支机构，在乌兰巴托设立巴彦淖尔商贸联络办。实施口岸“三互”大通关改革试点，推广应用国际贸易“单一窗口”，让进出口更加方便快捷。

　　抓好重点领域改革。进一步深化“放管服”改革，重点在“证照分离”和网上审批上取得突破性进展。推广“马上办”“最多跑一次”以及不见面的网上审批、事中事后综合监管等做法，在原来110项公共服务事项的基础上，再推进240项进驻政务服务中心，实现应进全进，为群众提供更集中、更简便、更快捷、更优质的服务。严格执行环保、能耗、质量等法律法规和标准，完成自治区下达的化解落后产能任务。综合运用土地、金融、棚改货币化安置等政策，化解商品房库存80万平方米。加快农村牧区综合改革，全面完成土地确权，启动实施集体产权制度改革，统筹推进“三权分置”改革，开展土地经营权和温室、圈舍等资产抵押贷款工作，深化农垦体制后续改革。继续抓好公立医院、综合行政执法等改革，分类推进事业单位改革，推动试点改革和自主探索改革。

　　提高对外开放水平。以建设对蒙开放先行区为目标，推动口岸自治区级重点开发开放试验区、边境经济合作区建设，实施通关区扩能升级工程，建成“七进七出”矿产能源通道和环保型仓储设施，争取启动跨境铁路和重载公路建设，力争口岸过货量达到3000万吨。设立俄蒙商品直销店，开通与蒙古国南戈壁省跨境航线，加强教育医疗、文化体育、商贸物流等领域深度合作。申建内蒙古国检试验区，完成乌拉特出入境检验检疫局、乌拉特海关、甘其毛都边检站迁址和更名工作，提高对外开放管理服务能力。深化区域经济合作，积极承接发达地区产业转移，努力实现共赢发展。

　　（八）提升城乡建设管理水平。坚持民生城建理念，不断改善城乡人居环境，让群众生活更方便、更舒心、更美好。

　　加强城镇规划建设管理。重点实施六大行动。一是规划引领行动。按照建设现代化生态田园城市的要求，修编城市整体规划、双河区分区规划、口岸集镇规划，完成“城市双修”、乡村振兴规划编制，积极推进“多规合一”。抓好包银高铁沿线重要节点、重点地段规划设计。要把富有独特魅力的民族地域文化和优秀传统文化融入城市规划设计，做好历史建筑确定和保护工作。二是道路畅通行动。新改建城镇道路48公里，改造背街巷道32万平方米，打通临河区塞北街、金川大道北延、杭锦后旗塞上东路、乌拉特前旗白彦花大街等18条“断头路”。大力发展公共交通，调整优化公交线路，全市新增电动公交车50辆，新能源公交车占比达60%以上。探索采用民营公助、PPP等方式，推进临陕客运班线公交化改造。针对不同时段，科学制定分流措施，缓解城市拥堵问题。三是绿化升级行动。注重城镇绿化规划设计和景观效果，坚持乔灌草结合、花果景搭配，见缝插绿、拆墙透绿、出门见绿，新增园林绿地面积3000亩。四是旧城改造行动。开工棚户区改造12877套，基本建成5554套，改造老旧小区230万平方米、平房区150万平方米，新增集中供热面积240万平方米、天然气用户6000户，新改建地下管网150公里、厕所125个，城区实现水冲式厕所全覆盖。完成临河区人民医院迁址工作，启动旧址改造，完善中心城区公共服务功能。采取有力措施，集中处理一批群众反映强烈的房地产“烂尾楼”“半拉子”工程，实现所有逾期回迁房交付使用，彻底化解回迁矛盾。用三年时间全面完成临河区北边渠综合改造工程，年内实施棚户区改造2838套。五是双河区建设行动。更多运用市场手段推动双河区建设，重点发展生态旅游、文体教育、健康养老、会展服务等产业，推动市委党校新校区、内蒙古美术职业学院新校区、电力综合服务中心、敕勒大街等项目竣工投用，开工建设三封路、高阙路等项目。六是管理提升行动。全面推行“街长制”管理模式，实现城镇精细化、网格化管理。推动机关单位停车场向社会全面开放，有效缓解停车难题。依法治理居民小区私搭乱建、破墙开店、乱堆乱放等问题，提高物业管理服务水平。

　　巩固提升农村牧区建设成果。启动农村牧区人居环境整治三年行动，完成100个中心村环境综合治理，建成10个农村集镇污水处理厂，推行农村垃圾“户投放、村收集、镇转运、县处理”，确保1万户农牧民使用上清洁的水冲厕所。全面推进“四好”农村牧区公路建设，新增公路里程500公里。实施偏远农牧区用电升级改造1763户，改善14万户农牧民饮水质量。开展美丽宜居乡镇、特色景观旅游名镇、特色小镇、绿色村庄等创建活动。做好嘎查村“两委”换届工作。广泛开展文明村镇、文明家庭、“干净人家、精巴媳妇”创建和家风家训评议等活动，培育文明乡风、良好家风、淳朴民风。

　　（九）着力保障改善民生和创新社会治理。群众利益无小事，一枝一叶总关情。我们要紧盯民生短板和薄弱环节，做好普惠性、基础性、兜底性民生建设，推进基本公共服务均等化，保障群众合法权益，促进社会公平正义。

　　多渠道促进就业和增收。全面落实就业创业扶持政策，抓好市创业创新示范基地和创业园建设。提供全方位公共就业服务，加强定向式、订单式培训，促进高校毕业生、农牧民工等重点群体就业创业，农村牧区转移就业12万人次，扶持2000人成功创业。深入挖掘增收潜力，释放农村牧区改革红利，落实惠农惠牧补贴政策，让农牧民的钱袋子实实在在鼓起来。

　　提高社会保障水平。全面实施全民参保计划，调整企业和机关事业单位退休人员基本养老金，提高城乡居民基础养老金最低标准。适度提高城乡低保标准和优抚补助标准，加强对特困人员、残疾人、优抚对象等群体的兜底保障。抓好城镇困难群体的脱困帮扶工作。投资1.8亿元，建成老年养护院4个，新增床位1720张。规范殡葬服务业。探索建立多主体供应、多渠道保障、租购并举的住房保障制度。落实农牧民工工资支付保障制度，着力解决工资拖欠问题，维护农牧民工合法权益。

　　办好人民满意教育。实施第三期学前教育行动计划。推进义务教育优质均衡发展和城乡一体化发展，推行校长教师交流轮岗，开展教育现代化强县创建活动。推动普通高中优质特色多样化发展，提高校园足球特色学校建设水平。招聘257名优秀人才充实教师队伍，优化教师队伍结构。投资7500万元，新改扩建幼儿园13所；投资2.3亿元，建成临河区水源路九年一贯制学校；投资2.1亿元，建成市职业技术培训实训基地（西贝美食艺术学校）和市示范性综合实践基地。河套学院新校区7月底前搬迁入驻，设立生态与资源工程系，促进高等教育内涵式发展。完成老年大学迁址工作，彻底改善办学条件。

　　加强科技创新。积极创建国家农业高新技术产业开发区，争取设立中国农科院区域研究中心，推动与自治区农牧业科学院等科研机构开展全面战略合作。发挥企业技术创新主体作用，积极设立研发中心，新增国家高新技术企业5家以上。加大产学研用合作力度，完成重大科技成果转化20项，新增授权专利180件以上。认真落实全市人才引进和培养实施办法，提高人才待遇，增加科研经费，引进高层次和急需紧缺人才。

　　推动文化繁荣发展。认真落实意识形态工作责任制，加强阵地建设和管理。深入挖掘河套文化内涵，筹划全流域黄河文化博物馆建设，推动阴山岩刻申遗工作。实施文化惠民工程，抓好图书馆、报刊亭建设，支持乌兰牧骑发展，创作一批纪念改革开放40周年文艺精品，开拍大型革命历史电视剧《鸿雁》。开工建设河套文化旅游产品创作园。办好河套文化艺术节、“一带一路”国际岩刻艺术作品“双年展”、中国岩刻丝路行——阴山岩刻国际巡展。推进学校体育场馆节假日向社会免费开放，建成乌拉特前旗、磴口县全民健身中心，实现全民健身中心旗县区全覆盖。

　　推进健康巴彦淖尔建设。全面推进分级诊疗和家庭医生签约服务，县域内就诊率达到90%以上，签约服务率达到40%以上。加大全科医生培养力度。推动“互联网+”创新医疗服务，促进医疗信息互联互通。扶持蒙中医药事业发展，提升妇幼健康服务机构标准化水平。加强农牧民健康管理。启动地区性重大疾病摸底调查工作。深入开展爱国卫生运动，启动创建国家卫生城市。大力推行“明厨亮灶”工程，改造提升餐饮企业1200家以上。加大食品药品安全监管力度，推进国家食品安全城市创建。

　　促进民族团结进步。全面贯彻党的民族政策，坚持民族区域自治制度，推进兴边富民行动，落实一线边民补助政策，大力发展民族教育、民族文化、民族体育事业，着力解决少数民族群众上学、就业、看病等问题，筑牢各族干部群众“三个离不开”和“五个认同”的思想根基，深入开展民族团结进步创建活动，争创全国民族团结进步示范市。

　　提高社会治理水平。深入推进警务云建设，健全完善立体化信息化社会治安防控体系，严厉打击暴力恐怖、涉枪涉暴和黄赌毒黑拐骗等违法犯罪活动。认真落实信访“1+7”制度，依法及时就地解决群众合理诉求，着力构建立体式群众诉求表达平台。全面落实安全生产责任制，坚决防止重特大安全生产事故发生。认真贯彻新修订的《宗教事务条例》，抓好双拥优抚、妇女儿童、统计等工作。

　　（十）加强和改进政府自身建设。打铁必须自身硬。我们一定要以志不求易、事不避难的决心和担当，不断提高执政能力水平，全心全意为人民服务。

　　我们要旗帜鲜明讲政治，牢固树立“四个意识”，增强“四个自信”，坚决维护以习近平同志为核心的党中央权威和集中统一领导，做到党中央提倡的坚决响应、党中央决定的坚决执行、党中央禁止的坚决不做。深入学习宣传贯彻党的十九大精神，自觉用习近平新时代中国特色社会主义思想武装头脑、指导实践、推动工作。

　　我们要坚持依法行政，始终把政府工作纳入法治化轨道，认真执行重大行政决策程序，着力加强合法性审查。坚持“三重一大”事项向市委常委会报告制度，自觉接受市人大及其常委会的依法监督、市政协的民主监督，做好建议提案办理工作。全面实施“七五”普法。支持法院、检察院依法行使职权，推进公安改革，维护社会公平正义。

　　我们要全面履职尽责，进一步提高执行力，把抓落实作为检验工作成效的重要标准，坚定“不折不扣”的执行意识，接受任务不讲条件，落实工作不找借口，杜绝议而不决、决而不行、行而无果。完善跟踪问效机制，对重点项目、重大事项、重要工作实行清单化管理，以工作目标倒逼工作进度，层层传导压力，逐级压实责任，确保各项工作落到实处。建立健全激励约束、容错纠错、督查问责机制，让敢于担当者放手放胆干事，让庸政懒政怠政者受到惩戒。

　　我们要加强学习和调查研究，加强对政策理论、专业知识的学习，加强对新技术、新产业、新业态、新模式的研究，学以致用，知行合一，切实把学习成果转化为谋划思路、指导实践、推动工作的具体行动。要大兴调查研究之风，既要到工作局面好和先进的地方总结经验，又要到困难较多、情况复杂、矛盾尖锐的地方研究问题，特别要对群众最关心、最期盼、最诟病、最苦恼、最痛恨的问题主动调研，切实把存在的矛盾和问题摸清搞准，使制定的政策措施更加符合实际、符合群众意愿。

　　我们要认真履行全面从严治党责任，严格执行中央八项规定实施细则、自治区党委配套规定，坚持不懈改作风转作风，坚决纠正“四风”新表现。坚持一切从实际出发，严肃纪律，切实把经济数据做实。深入推进“两学一做”学习教育常态化制度化，开展“不忘初心、牢记使命”主题教育，全面提升政府系统党建工作水平。加强审计监督，支持监察委员会工作，坚决整治损害群众利益的行为。

　　各位代表，刚刚闭幕的市委四届四次全会暨全市经济工作会议，提出了把巴彦淖尔建设成为绿色产业集聚区、生态文明示范区、乡村振兴样板区、对蒙开放先行区、美好生活共享区，建成现代化生态田园城市，实现塞上江南、绿色崛起。这一奋斗目标，反映了全市各族人民对巴彦淖尔美好前景的期盼。幸福都是奋斗出来的，我们要撸起袖子加油干，一步一个脚印把宏伟蓝图变为美好现实。

　　各位代表，时代赋予重任，实干铸就辉煌。让我们更加紧密地团结在以习近平同志为核心的党中央周围，在习近平新时代中国特色社会主义思想的指引下，在自治区党委、政府和市委的正确领导下，不忘初心，牢记使命，以永不懈怠的精神状态、一往无前的奋斗姿态，攻坚克难、干事创业，为全市各族人民生活得更加美好，为实现塞上江南、绿色崛起而不懈奋斗！
